# The FDA-Approved Drug Nelfinavir Inhibits Lytic Cell-Free but Not Cell-Associated Nonlytic Transmission of Human Adenovirus

**DOI:** 10.1128/AAC.01002-20

**Published:** 2020-08-20

**Authors:** Fanny Georgi, Vardan Andriasyan, Robert Witte, Luca Murer, Silvio Hemmi, Lisa Yu, Melanie Grove, Nicole Meili, Fabien Kuttler, Artur Yakimovich, Gerardo Turcatti, Urs F. Greber

**Affiliations:** a Department of Molecular Life Sciences, University of Zurich, Zurich, Switzerland; b Biomolecular Screening Facility, School of Life Sciences, Ecole Polytechnique Fédérale de Lausanne, Lausanne, Switzerland; c MRC Laboratory for Molecular Cell Biology, University College London, London, United Kingdom; d Artificial Intelligence for Life Sciences CIC, London, United Kingdom

**Keywords:** adenovirus death protein, antiviral agents, cell lysis, compound screening, drug repurposing, fluorescence imaging, membrane rupture, oncolytic virus, plaque assay, virus transmission

## Abstract

Adenoviruses (AdVs) are prevalent and give rise to chronic and recurrent disease. Human AdV (HAdV) species B and C, such as HAdV-C2, -C5, and -B14, cause respiratory disease and constitute a health threat for immunocompromised individuals. HAdV-Cs are well known for lysing cells owing to the E3 CR1-β-encoded adenovirus death protein (ADP). We previously reported a high-throughput image-based screening framework and identified an inhibitor of HAdV-C2 multiround infection, nelfinavir mesylate. Nelfinavir is the active ingredient of Viracept, an FDA-approved inhibitor of human immunodeficiency virus (HIV) aspartyl protease that is used to treat AIDS. It is not effective against single-round HAdV infections. Here, we show that nelfinavir inhibits lytic cell-free transmission of HAdV, indicated by the suppression of comet-shaped infection foci in cell culture. Comet-shaped foci occur upon convection-based transmission of cell-free viral particles from an infected cell to neighboring uninfected cells. HAdV lacking ADP was insensitive to nelfinavir but gave rise to comet-shaped foci, indicating that ADP enhances but is not required for cell lysis. This was supported by the notion that HAdV-B14 and -B14p1 lacking ADP were highly sensitive to nelfinavir, although HAdV-A31, -B3, -B7, -B11, -B16, -B21, -D8, -D30, and -D37 were less sensitive. Conspicuously, nelfinavir uncovered slow-growing round HAdV-C2 foci, independent of neutralizing antibodies in the medium, indicative of nonlytic cell-to-cell transmission. Our study demonstrates the repurposing potential of nelfinavir with postexposure efficacy against different HAdVs and describes an alternative nonlytic cell-to-cell transmission mode of HAdV.

## Abstract (precis)

Adenoviruses (AdVs) are prevalent and give rise to chronic and recurrent disease. Human AdV (HAdV) species B and C, such as HAdV-C2, -C5, and -B14, cause respiratory disease and constitute a health threat for immunocompromised individuals. HAdV-Cs are well known for lysing cells owing to the E3 CR1-β-encoded adenovirus death protein (ADP). We previously reported a high-throughput image-based screening framework and identified an inhibitor of HAdV-C2 multiround infection, nelfinavir mesylate.

## INTRODUCTION

Adenovirus (AdV) was first described in 1953 by Rowe and coworkers as a cytopathological agent isolated from human adenoids (1). More than 100 human AdV (HAdV) genotypes have since been characterized by molecular genetics or serology and grouped into seven species (2, 3). HAdV species A, F, and G replicate in the gastrointestinal tract; species B, C, and E replicate in the respiratory organs; and species B and D replicate in conjunctival cells of the eyes. Species B members have a broad tropism, including kidney and cells of the hematopoietic lineage (4–6). HAdV-caused illness can range from asymptomatic to lethal, especially in immunocompromised individuals (7–9). HAdV outbreaks are frequent in military training camps but also nursing homes, as recorded in recurrent outbreaks of HAdV-E4 and HAdV-B7 (5, 10–13). To counter the disease burden, an oral HAdV-E4/B7 vaccine was reintroduced, leading to a sharp decline in adenoviral disease among military recruits (5, 14, 15). In addition to recurrent HAdV outbreaks, novel HAdV variants emerge, with some of them causing pneumonia and death of the elderly with chronic diseases. One of these emerging HAdVs is the HAdV-B14 variant 14p1, also known as 14a (16–20). Furthermore, AdVs have the potential for zoonotic transmission (21). Cross-species infections of humans from either nonhuman primates or psittacine birds have been reported from the United States and China, respectively (22, 23). Despite the high prevalence (5, 24–26) and the broad use of AdVs as gene therapy vectors (27) as well as oncolytic viruses (28, 29), no FDA-approved specific anti-HAdV treatment is available to date. Clinically, HAdV infections are treated with ribavirin, cidofovir, or, more recently, brincidofovir, all of which inhibit viral DNA replication (30, 31).

HAdV particles have been well characterized. They have a double-stranded DNA genome of ∼36 kbp packaged into an icosahedral capsid of about 90 nm in diameter (32–35). The HAdV-C2 and -C5 replication cycle has been extensively studied, including entry, uncoating, replication, assembly, and egress from the infected cell (36–50). HAdV-C infects cells by binding to the coxsackievirus adenovirus receptor (CAR) and integrin coreceptors, followed by receptor-mediated endocytosis, endosomal lysis, and microtubule-motor-driven transport to the nucleus, where it uncoats DNA and delivers the DNA into the nucleus (38, 51–62). The first viral protein expressed is E1A, a multifunctional, intrinsically disordered protein controlling the transcriptional activity of all AdVs as well as many cellular promoters, thereby affecting the cell cycle, differentiation, transformation, and apoptosis (63–68). Viral early proteins besides E1A mediate immune escape, block the activation of proapoptotic pathways, and form nuclear viral DNA replication compartments. Late viral proteins give rise to mature progeny virions upon limited proteolysis of capsid proteins by the viral cysteine protease L3/p23 (69–71). Mature HAdV progeny is released upon the rupture of the nuclear envelope and plasma membrane, which facilitates rapid viral dissemination and plaque formation *in vitro* (72–74). The convection forces in the medium give rise to comet-shaped infection foci in cell cultures (72). Foci of infected cells are also found in tissue such as rat liver upon the intravenous inoculation of HAdV-C5 (75). Accordingly, acute HAdV infections trigger an inflammatory response, as shown in airways or conjunctiva of susceptible animals (2, 76). In contrast to lytic virus transmission, direct cell-to-cell transmission leads to round plaques, as shown with vaccinia virus (77–80).

The mechanisms of virus transmission are highly virus specific. They comprise nonlytic pathways involving secretory-endocytic circuits, multivesicular or autophagic membrane processes, cellular protrusions, or transient breaches of membrane integrity (80–84). In contrast, lytic egress pathways further involve the destabilization of cellular membranes by viral and host factors, often tuned by the cytoskeleton (37, 85–88). HAdV-C2 controls lytic cell death by the adenovirus death protein (ADP), also known as 11.6K, as concluded from genetic and overexpression studies (73, 74). ADP is a type III membrane protein transcribed from the CR1-β region in the immunoregulatory E3a locus. All HAdV-C members harbor homologous E3a CR1-β sequences (e.g., 10.5K in HAdV-C5). Other HAdV species differ in their E3 regions, however (89–91). The N terminus of ADP is luminal, and the C terminus protrudes into the cytosol (92). Following posttranslational modifications, ADP is transported to the inner nuclear membrane, where the N terminus is intruding into the nucleus (93). At late stages, when capsid assembly in the nucleus has commenced, ADP expression is boosted (94, 95). The mechanism of host cell lysis is still unknown, although necrosis-like, autophagic, and caspase activities have been implicated (96–99).

Here, we report that nelfinavir mesylate (nelfinavir for short) is an effective inhibitor of HAdV lytic egress. The procedure leading to the identification of nelfinavir is described in another study using an imaging-based, high-content screen of the Prestwick Chemical Library (PCL) comprising 1,280 mostly clinical or preclinical compounds (100, 101). Nelfinavir is the off-patent active pharmaceutical ingredient of Viracept, an FDA-approved drug that inhibits the human immunodeficiency virus (HIV) protease (102). The work here documents the repurposing potential of nelfinavir, which is effective against a spectrum of HAdV types in a postexposure manner. Nelfinavir is partly, but not exclusively, active against ADP-encoding HAdV types and uncovers the appearance of round plaques, which arise upon nonlytic cell-to-cell viral transmission.

## RESULTS

### Nelfinavir is a nontoxic, potent inhibitor of HAdV-C multicycle infection.

**FIG 1:**
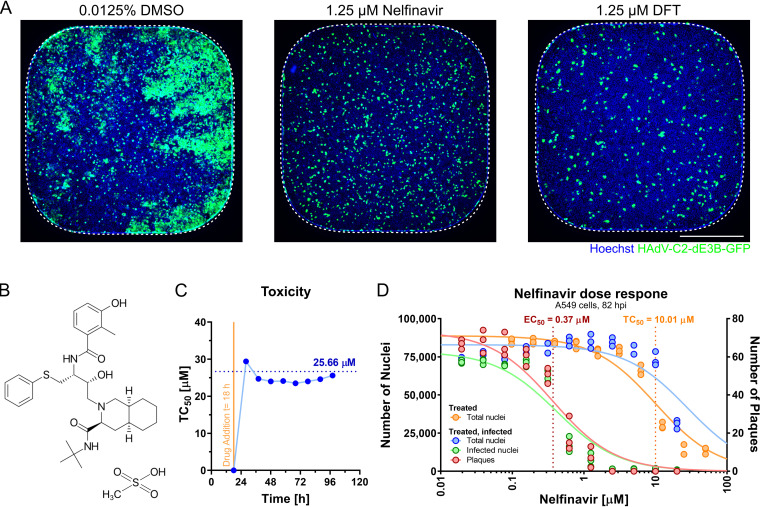
The small molecule nelfinavir is a potent inhibitor of HAdV-C infection. (A) Representative 384-well epifluorescence microscopy images of cells treated with DMSO (left), nelfinavir (center), and DFT (right) and infected with HAdV-C2-dE3B-GFP for 72 h. Dotted lines indicate the well outline. Bar = 5 mm. (B) Structural formula of nelfinavir mesylate. (C) The half-maximal toxicity (TC_50_) in uninfected A549 cells was determined by nelfinavir dose-response impedance measurements at different times of drug treatment. The *x* axis indicates the times after cell seeding as well as drug addition. Impedance was recorded at intervals of 15 min using xCELLigence reporting on the cell number and cell adhesion to the electrode-coated wells. The raw CI data are available in Fig. S1 in the supplemental material. (D) Separation of effect (EC_50_) (plaque numbers) and toxicity (TC_50_) (nucleus numbers) of nelfinavir in A549 cells at 82 hpi based on data from four technical replicates.

A recent paper describes a full-cycle, image-based screen of 1,278 out of 1,280 PCL compounds against HAdV-C2-dE3B-GFP, where clopamide and amphotericin B were excluded due to precipitation during acoustic dispension into the screening plates (100). The screen was conducted in adenocarcinomic human alveolar basal epithelial (A549) cells at a 1.25 μM compound concentration and identified nelfinavir, aminacrine, dequalinium dichloride, and thonzonium bromide as hits (see Table S1 in the supplemental material). Nelfinavir (CAS number 159989-65-8) strongly inhibited plaque formation at nanomolar concentrations, comparably to the known HAdV nucleoside analogue inhibitor 3′-deoxy-3′-fluorothymidine (DFT) (Fig. 1A and B). Dequalinium dichloride, aminacrine, and thonzonium bromide were excluded from further analyses due to toxicity (100) and potential mutagenic effects (103). Long-term incubations of uninfected A549 cells with nelfinavir for up to 115 h showed median toxicity (concentration causing 50% toxicity [TC_50_]) values of 25.7 μM, as determined by cell impedance measurements using xCELLigence (Fig. 1C). xCELLigence measures the impedance of electrical currents imposed by cell adherence to gold-plated microelectrodes implanted in culture wells. Impedance is expressed as a cell index (CI), a unitless parameter proportional to the cell number, cell size, and cell adherence. For raw CI profiles, see Fig. S1A in the supplemental material. CI measurements were consistent with data from presto_blue assays and cell numbers determined by counting nuclei (Table S1). This was in agreement with previous reports and acceptable side effects in clinical use against HIV (102, 104). The median therapeutic index (TI_50_) of nelfinavir was 27.1 (Fig. 1D), as determined by the ratio between the concentration yielding a 50% loss of cell nuclei (TC_50_ = 10.01 μM) and the effective concentration yielding 50% inhibition (EC_50_) of fluorescent-plaque formation (EC_50_ = 0.37 μM). The data indicate that nelfinavir is an effective, nontoxic inhibitor of HAdV-C2 multicycle infection.

### Nelfinavir does not affect single-round infection.

**FIG 2:**
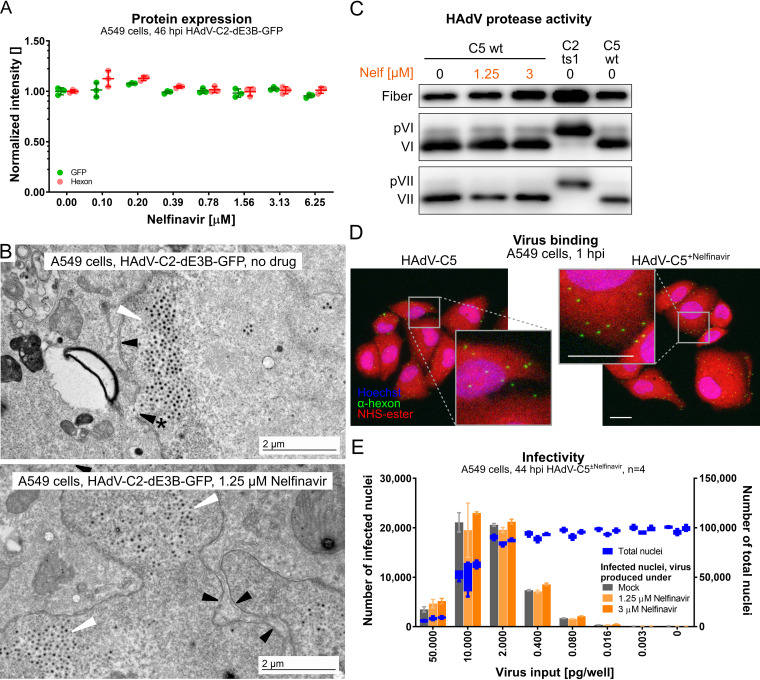
Nelfinavir does not affect early or late steps of HAdV-C infection. (A) No effect of nelfinavir on the expression of CMV-GFP or the late viral protein hexon in HAdV-C2-dE3B-GFP-infected A549 cells. For each of the four biological replicates, data points represent the mean median nuclear intensities per well normalized to the mean median nuclear intensities of the DMSO-treated wells. Epifluorescence microscopy images were segmented and analyzed using CellProfiler. (B) Representative transmission EM images of late-stage HAdV-C2-dE3B-GFP-infected A549 cells at 41 hpi reveal viral particles inside the nucleus in both DMSO-treated and nelfinavir-treated cells (white arrowheads). Black arrowheads indicate the nuclear envelope, and the arrowhead with * points to a rupture. (C) Nelfinavir does not affect the maturation of HAdV-C5, as indicated by fully processed VI and VII proteins in purified particles grown in the presence of nelfinavir. Note that HAdV-C2-*ts*1 lacking the L3/p23 protease contains the precursor capsid proteins of VI and VII (pVI and pVII). (D) HAdV-C5 grown in the presence of nelfinavir (HAdV-C5^+Nelfinavir^) binds to naive A549 cells similarly to HAdV-C5 from control cells. Cells were incubated with the virus at 4°C for 1 h and fixed with PFA. Images are maximal projections of confocal z stacks and also show zoomed-in views (gray squares). Bars = 20 μm. (E) Particles produced in the presence of nelfinavir are fully infectious. A549 cells were inoculated with purified HAdV-C5 and incubated in the absence or presence of nelfinavir for 44 hpi. Shown are data for infection analyses by antihexon immunofluorescence staining and cell numbers derived from Hoechst staining. Bars represent means from four technical replicates. Error bars indicate standard deviations.

We first tested if nelfinavir affected viral protein production. HAdV-C2-dE3B-GFP-infected A549 cells were analyzed for green fluorescent protein (GFP) under the control of the immediate early cytomegalovirus (CMV) promoter and the late protein hexon expressed after viral DNA replication at 46 h postinfection (hpi). The results indicate that nelfinavir had no effect on GFP or hexon expression at the tested concentrations, while the formation of fluorescent plaques was completely inhibited (Fig. 1D and Fig. 2A). This result was in agreement with the notion that nelfinavir did not affect the replication of the HAdV-C5 genome, as determined by titration of cell-associated infectious particles (105). We next examined if nelfinavir affected the formation of viral particles. Transmission electron microscopy (EM) of HAdV-C2-dE3B-GFP-infected cells revealed large numbers of virions in the nuclei of nelfinavir-treated and untreated cells (Fig. 2B). This result was in agreement with the observation that the nuclei of nelfinavir-treated cells expanded in area over time and were indistinguishable from those of control cells (Fig. S1B).

To test if nelfinavir affected virion maturation, we analyzed purified virions by SDS-PAGE and Western blotting against the precursor VI (pVI)/VI and pVII/VII proteins using previously characterized antibodies. There was no evidence for an increase of pVI or pVII in HAdV-C5 from nelfinavir-treated cells, in contrast to temperature-sensitive 1 (*ts*1) particles, which lack the L3/p23 protease due to the point mutation P137L in p23 (106) (Fig. 2C). This shows that nelfinavir did not affect the proteolytic maturation of the virus by the L3/p23 cysteine protease. In accordance, purified HAdV-C5 from nelfinavir-treated cells attached to naive A549 cells and gave rise to viral gene expression as effectively as control HAdV-C5 particles (Fig. 2D and E). Together, these results indicate that nelfinavir does not affect the production of infectious virions in single-round infections.

### Nelfinavir inhibits HAdV-C egress.

**FIG 3:**
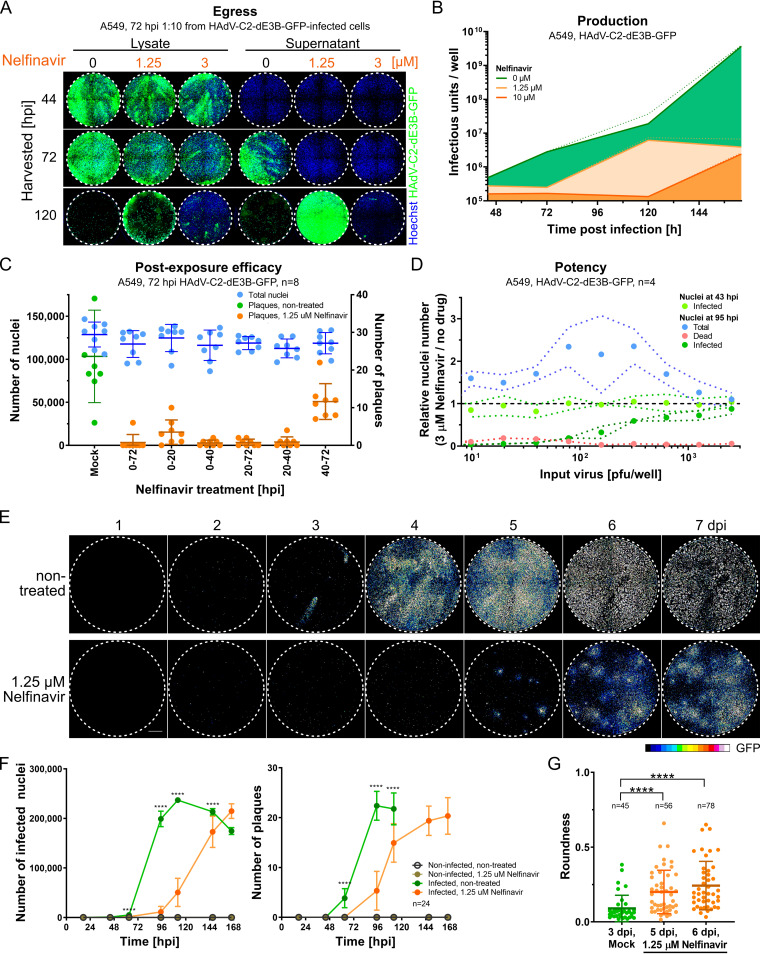
Nelfinavir is a postexposure inhibitor of HAdV-C egress. (A) A549 cells were imaged at 3 days postinoculation with 1:10-diluted cell lysates (left) or supernatants (right) from nelfinavir-treated or control A549 cells, which had been infected with HAdV-C2-dE3B-GFP for the indicated times (harvested [hpi]). The results show delayed viral progeny release into the supernatant of nelfinavir-treated cells. Nuclei are shown in blue, and infection markers are shown in green (GFP). (B) Released and cell-associated progeny from HAdV-C2-dE3B-GFP-infected A549 cells treated with nelfinavir or DMSO (green), as determined by titration on A549 cells in a 12-well assay format. Lines indicate mean slopes, and dotted lines indicate standard errors. Linear regression of data from three biological triplicates is shown. (C) Time-resolved emergence of plaques in HAdV-C2-dE3B-GFP-infected A549 cells treated with 1.25 μM nelfinavir. Data points represent results from one of eight technical replicates. Colored vertical lines indicate the means, and error bars indicate the standard deviations. (D) The inhibitory effect of nelfinavir on HAdV-C2-dE3B-GFP spread is dependent on the amount of input virus during initial infection. The number of infected GFP-positive cells is shown at 3 μM nelfinavir relative to the mean number of solvent-treated cells infected with the corresponding dosage. Note that the number of infected cells at 43 hpi is not affected by nelfinavir treatment. Data points represent means from four technical replicates. Dotted lines indicate standard deviations. (E) Treatment of HAdV-C2-dE3B-GFP-infected A549 cells with 1.25 μM nelfinavir suppresses comet-shaped plaques and reveals slow-growing quasiround plaques. Viral GFP expression levels are shown as 16-color look-up table (LUT). Bar = 1 mm. (F) Treatment with 1.25 μM nelfinavir inhibits HAdV-C2-dE3B-GFP infection of A549 cells by slowing plaque formation. Data points represent means from 24 technical replicates, including the well shown in the micrographs of panel D. Error bars indicate standard deviations. Statistical significance of drug-treated versus nontreated cells was derived by the Kolmogorov-Smirnov test, with a *P* value of <0.0001 (****). (G) The delayed HAdV-C2-dE3B-GFP plaques in the presence of 1.25 μM nelfinavir are significantly rounder than control plaques, as indicated by a Kolmogorov-Smirnov test. Data points indicate plaque regions in the well center harboring a single peak region. Shown is a summary of data from 24 technical replicates, including the well shown in the micrographs of panel D. Regions consisting of at least 5 infected cells (≥1,500 μm^2^) were considered a plaque. Plaque morphologies in control wells could not be quantified later than 3 dpi due to rapid virus dissemination. For plaques from DMSO-treated cells at 3 dpi compared to nelfinavir-treated ones at 5 dpi, the approximate *P* value was <0.0001 (****). For DMSO-treated plaques at 3 dpi versus nelfinavir-treated plaques at 6 dpi, the approximate *P* value was <0.0001 (****). Statistical significance was determined by a Kolmogorov-Smirnov test.

We investigated the kinetics of HAdV-C2-dE3B-GFP production and release into the supernatant. Supernatants and whole-cell lysates of treated and nontreated infected cells were harvested at different time points (Fig. 3A). At 44 hpi, cell lysates of nelfinavir-treated and control cells showed similar infectivities but did not yet release virus into the supernatant, as shown by titration on naive A549 cells. At 72 or 120 hpi, control cells, but not nelfinavir-treated cells, had released virus into the supernatant. Notably, the viral titer in the supernatant of control cells at 120 hpi was so high that nearly all the cells in the indicator plates dissociated from the plates. The difference in the infectious load was confirmed by titration of supernatants from separate time course experiments at three different concentrations of nelfinavir (Fig. 3B). At 7 days postinfection (dpi), a dosage of 1.25 μM reduced the total yield of infectious particles in the supernatant by 3 orders of magnitude, underscoring the potency of nelfinavir in blocking the dissemination of HAdV-C-dE3B-GFP. Moreover, nelfinavir limited HAdV-C2 transmission when added as late as 40 hpi (Fig. 3C). These findings indicate that nelfinavir impairs the egress of progeny from the host cell.

We next assessed the potency of nelfinavir against HAdV-C2 transmission by quantifying the number of nuclei, which normally decreases due to lytic virus replication. Nelfinavir (3 μM) robustly reduced the number of dead cells and strongly reduced the number of infected cells at up to 100 PFU/well (Fig. 3D). Remarkably, HAdV-C2-dE3B-GFP formed delayed plaques in the presence of nelfinavir starting at 4 dpi (Fig. 3E and F). These late plaques showed a strikingly round morphology, which was calculated to be significantly different from the comet-shaped plaques early in infection of control cells (Fig. 3G). The direction of the comet tail of lytic plaques can be aligned by tilting the incubation plate (72). Thereby, the cell monolayer is positioned nonorthogonally to the vector of thermal convection flux of the liquid cell culture medium. While the direction of the comet-shaped plaques could be aligned using this method in nontreated infections, the late nelfinavir-treated plaques remained mostly round (Fig. S2A to C). Moreover, there was no correlation between the size of the plaques and their roundness irrespective of nelfinavir up to 7 dpi, demonstrating that the round plaques did not change morphology over time (Fig. S2D). Collectively, the data indicate that virus transmission in the presence of nelfinavir is not driven by the bulk current of cell-free medium.

### HAdV inhibition by nelfinavir depends on ADP.

**FIG 4:**
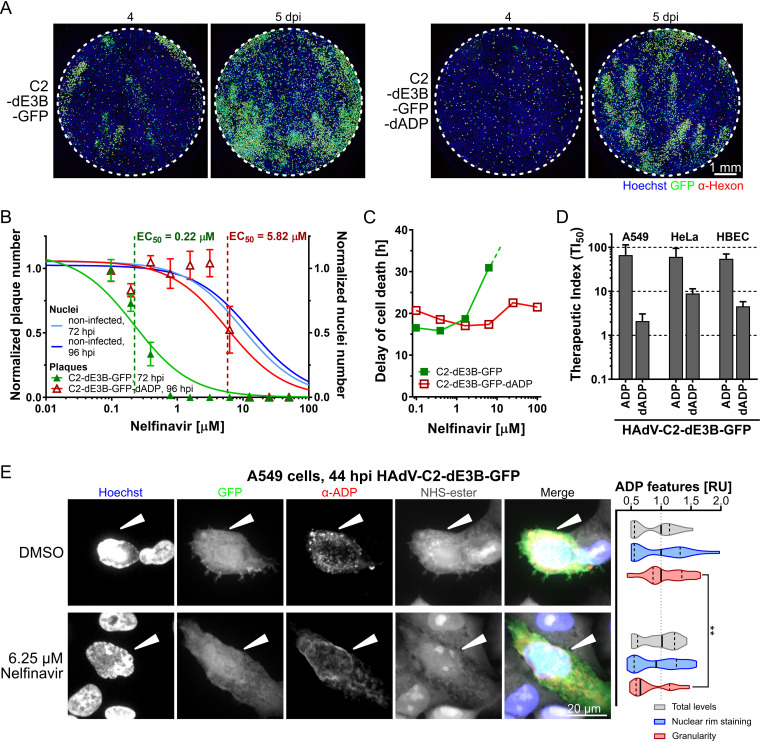
ADP contributes to the inhibitory effect of nelfinavir against HAdV-C. (A) The deletion of ADP from HAdV-C2-dE3B-GFP delays plaque formation in A549 cells by 1 day but does not change plaque shape. Cells were infected with 1.1 × 10^5^ VP/well. (B) The deletion of ADP from HAdV-C2-dE3B-GFP reduces the antiviral effects of nelfinavir in A549 cells, with an EC_50_ of 5.82 compared to 0.22 μM for the parental virus. HAdV-C2-dE3B-GFP infection was quantified at 72 hpi, and ADP deletion mutant infection was quantified at 96 hpi. Plaque numbers per well were normalized to the mean DMSO control value. Numbers of nuclei in noninfected, treated wells were normalized to the mean for the DMSO control. Data points represent means from four technical replicates. Error bars indicate standard deviations. EC_50_ values were derived from nonlinear curve fitting. For detailed information and statistics, see Table S2 in the supplemental material. (C) The delay of dell death was calculated from the highest mean cell index (CI) and its half-maximum for each treatment (means from two technical replicates). For HAdV-C2-dE3B-GFP-infected A549 cells treated with 25 μM nelfinavir, the measurement was aborted due to overgrowth causing cytotoxicity before the maximal cell index was reached. Treatment with 100 μM nelfinavir was toxic. (D) TI_50_ derived from the ratio of the nelfinavir concentration causing 50% toxicity (TC_50_) and the concentration leading to a 50% reduction in plaque numbers per well (EC_50_). Results from different cancer and primary cells are shown for HAdV-C2-dE3B-GFP and HAdV-C2-dE3B-dADP lacking ADP. For detailed information and statistics, see Table S2. (E, left) Representative high-magnification confocal images of HAdV-C2-dE3B-GFP-infected A549 cells at 44 hpi showing the effect of nelfinavir on ADP localization. ADP was stained by immunofluorescence with a rabbit anti-HAdV-C2-ADP_87–101_ antibody. Cells were stained using NHS-ester. White arrowheads highlight infected cells. Nuclei are in blue. Images are maximal projections of 30 z planes with 0.5-μm z steps. (Right) Relative units (RU) of total ADP expression, localization to the nuclear rim, and granularity normalized to the mean values from DMSO-treated control cells. The data set is comprised of 20 nelfinavir-treated infected cells and 23 control cells. Solid lines indicate medians, and dotted lines indicate the 5 to 95% quantiles. The Kolmogorov-Smirnov test indicated an ADP granularity *P* value of 0.0019 (**).

ADP is expressed at high levels late in infection and enhances cell lysis (94, 107). To test if ADP was required for nelfinavir inhibition of lytic spread, we generated an ADP-deleted HAdV-C2-dE3B-GFP mutant, HAdV-C2-dE3B-GFP-dADP. The mutant completely lacks ADP expression, as indicated by immunofluorescence and Western blot experiments (Fig. S3A and B). HAdV-C2-dE3B-GFP-dADP formed particles indistinguishable from those of HAdV-C2-dE3B-GFP, as indicated by negative-stain EM (Fig. S3C). HAdV-C2-dE3B-GFP-dADP showed a delayed onset of plaque formation by about 1 day compared to HAdV-C2-dE3B-GFP (Fig. 4A). These data are in agreement with those of previous kinetic studies with the ADP deletion mutant HAdV-C dl712 (108) (see also Fig. S3A in the supplemental material). HAdV-C2-dE3B-GFP-dADP plaques were comet shaped, albeit their comet heads appeared larger and denser (Fig. 4A). While the parental virus was highly sensitive to nelfinavir, HAdV-C2-dE3B-GFP-dADP required much higher concentrations of the compound to show inhibition of plaque formation (Fig. 4B; Table S2). In accordance, the ADP-deleted virus induced cell death independent of nelfinavir, unlike the ADP-expressing virus, as concluded from cell impedance measurements with xCELLigence (Fig. 4C; Fig. S3D and E). Finally, HAdV-C2-dE3B-GFP-dADP exhibited a strongly diminished separation of antiviral efficacy from toxicity, as indicated by reduced TI_50_ values compared to the parental virus, for example, 2.1 versus 66.8 with A549 cells, 8.9 versus 61.0 with HeLa cells, and 4.6 versus 55.2 with human bronchial epithelial cells (HBECs) (Fig. 4D). These effects were in agreement with those from similar experiments performed with the previously described ADP knockout mutant dl712 and the parental virus rec700, an HAdV-C5/2 hybrid virus (107, 109). The data are shown in Fig. S3F to H in the supplemental material. Together, these results show that the selective antiviral effects of nelfinavir are more cell type dependent in the case of HAdV lacking ADP than in ADP-expressing viruses, and the effects are comparatively small for viruses lacking ADP.

Finally, we performed immunofluorescence experiments with HAdV-C2-dE3B-GFP-infected A549 cells at 44 hpi (Fig. 4E). Under nonperturbed conditions, ADP accumulated in cytoplasmic foci and the nuclear envelope. Nelfinavir treatment did not affect the overall ADP expression levels or the amount of ADP in the nuclear periphery, including the nuclear envelope, but completely abolished the cytoplasmic ADP foci as indicated by granularity quantifications (Fig. 4E, right graph). Intriguingly, Tollefson and coworkers observed previously that ADP lacking luminal O-glycosylation sites did not localize to large cytoplasmic granules, and the corresponding HAdV-C mutant pm734.4 was nonlytic (108). We speculate that the localization of ADP in cytoplasmic organelles such as Golgi compartments, where O-glycosylation occurs (110), could enhance the cell lytic function of ADP. Together, the data show that ADP is a major susceptibility factor for the inhibition of the spread of HAdV-C infection by nelfinavir.

### A round nonlytic plaque phenotype in HAdV-C infection.

**FIG 5:**
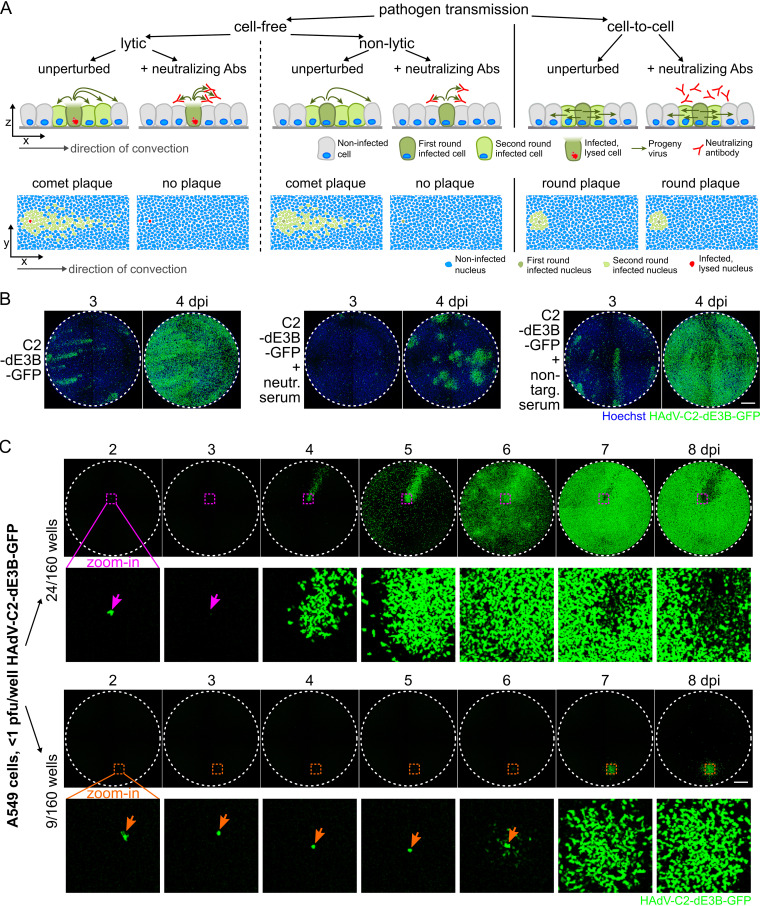
Round-plaque phenotypes in the presence of neutralizing anti-HAdV-C2 antibodies and in unperturbed HAdV-C2 infections. (A) Schematic overview of pathogen transmission routes in cell cultures. Cell lysis kills the donor cell and releases progeny, while nonlytic egress preserves the infected donor cell. Convection in the medium leads to long-distance, comet-shaped plaques, and cell-free virus transmission is susceptible to neutralizing antibodies (Abs). In contrast, direct cell-to-cell spread of the virus gives rise to symmetric slow-growing plaques resistant to neutralizing antibodies. Axes indicate side or top-down views. (B) Inhibition of cell-free HAdV-C2-dE3B-GFP transmission by anti-HAdV-C2/5 neutralizing serum. Nuclei are shown in blue. (C) Infection of A549 cells with limiting amounts of HAdV-C2-dE3B-GFP (<1 PFU/well; 9 to 75 VP/well) in 160 wells gives rise to 33 single plaques/well. Twenty-four wells contained GFP-positive comet-shaped plaques (top), and nine developed delayed round plaques (bottom). Dashed colored squares indicate magnified regions of the first-round infected cell below. An infected cell leading to a comet-shaped plaque (top, pink arrows) lyses at 3 dpi, as indicated by the loss of the GFP signal. An infected cell giving rise to a round plaque (bottom, orange arrows) remains GFP positive. Bar = 1 mm.

Viruses are transmitted between cells by three major mechanisms, cell free through the extracellular medium, directly from cell to cell, or in an organism by means of infected motile cells or fluid flow in blood or lymphoid vessels. This can result in far-reaching or mostly local virus dissemination (for a simplified cartoon, see Fig. 5A). In cell culture, HAdV-C transmission from a lytic infected cell (staining propidium iodide [PI] positive) yields comet-shaped infection foci due to convective passive mass flow in the cell culture medium (72, 101), consistent with lytic HAdV-C infection (74, 107). In accordance, neutralizing antibodies against HAdV-C2 added to the cell culture medium suppressed the comet-shaped plaques of HAdV-C2-dE3B-GFP and yielded confined, predominantly round infection foci at 4 dpi, akin to nelfinavir-treated infections (Fig. 5B).

To test if round infection foci (plaques) occurred in regular HAdV-C2-dE3B-GFP infections, we analyzed A549 cells infected with <1 PFU per well in 160 wells up to 8 dpi. Thirty-three wells developed a single plaque. Twenty-four of them were fast-emerging comet-shaped plaques, of which the donor cell (indicated by the pink arrows) disappeared at between 2 and 3 dpi (Fig. 5C, top). In contrast, nine wells developed delayed round plaques starting at 6 dpi (Fig. 5D, bottom). In all these cases, the original infected cell (orange arrows) remained GFP positive and apparently viable, and the surrounding cells gradually became infected. These data suggest that HAdV-C2 utilizes both lytic and nonlytic transmission, with the former involving cell-free transmission and the latter involving cell-associated transmission.

### Nelfinavir has a broad anti-HAdV spectrum.

**FIG 6:**
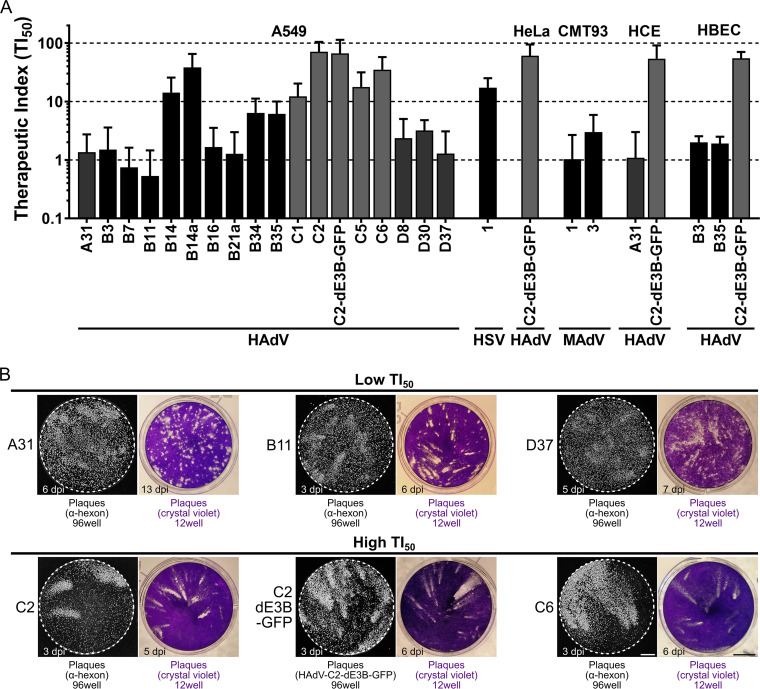
Susceptibility of HAdV to nelfinavir correlates with plaque shape. (A) TI_50_ calculated from the ratio of the nelfinavir concentration causing 50% toxicity (TC_50_) and the concentration leading to 50% plaque reduction (EC_50_). Different HAdVs, mouse adenoviruses (MAdV), and herpes simplex virus 1 (HSV-1) were tested in different cancer and primary cell lines. For detailed information and statistics, see Table S2 in the supplemental material. (B) Representative microscopic and macroscopic plaque morphologies of nelfinavir-sensitive and -insensitive HAdV types. Grayscale images show plaques based on epifluorescence microscopy of hexon immunostaining or GFP expression in A549 cells (96-well format) (bar = 1 mm). Colored images show plaques visualized by crystal violet staining in A549 cells (12-well format) (bar = 5 mm).

We finally assessed the inhibition breadth of nelfinavir against various HAdV types from species A, B, C, and D in different human cell lines as well as mouse adenovirus 1 (MAdV-1) and MAdV-3 in mouse rectum carcinoma CMT93 cells. To balance statistical significance and automated plaque segmentation, we first determined the optimal amount of inoculum and duration of infection for each virus and cell line. The resulting TI_50_ values of nelfinavir were heterogeneous for different HAdV types, as determined in A549 cells (Fig. 6A; for details, see Table S2 in the supplemental material), while all the tested HAdV-C types as well as HAdV-B14 showed high TI_50_s (>10) ranging from 12.22 (HAdV-C1) to 71.09 (HAdV-C2). Members of HAdV species A and D and most of the HAdV-B types showed intermediate (2 to 10) to low (<2) nelfinavir susceptibility, notably HAdV-B7 and -B11 with TI_50_s of <1. MAdV-1 and -3 also showed low susceptibility. Noticeably, a high susceptibility of HAdV-C was consistently observed in human lung epithelial carcinoma (A549) cells, human epithelial cervix carcinoma (HeLa) cells, immortalized primary normal human corneal epithelial (HCE) cells, as well as normal HBECs. The corresponding TI_50_ values were in the same range as those for herpes simplex virus 1 (HSV-1), for which nelfinavir was reported to be an egress inhibitor (105, 111, 112).

We finally examined the plaque morphologies in nonperturbed infections by immunofluorescence staining of the late proteins VI and hexon as well as by microscopic analyses of crystal violet-stained dishes for classical plaques (Fig. 6B). Viruses that were highly susceptible to nelfinavir (exhibiting high TI_50_ values) formed exclusively comet-shaped plaques. Viruses with low TI_50_ values, such as A31, B11, or D37, had a high fraction of round plaques even when infected with >1 PFU/well. This demonstrates that the slowly growing round infection foci observed by fluorescence microscopy gave similarly shaped lesions due to cytotoxicity, akin to the lytic comet-shaped foci. We conclude that HAdV types employ lytic cell-free and nonlytic cell-to-cell transmission modes and give rise to different plaque phenotypes.

## DISCUSSION

A phenotypic screen of the PCL identified nelfinavir as a potent postexposure inhibitor of HAdV-C2-dE3B-GFP plaque formation in cell culture (100). Nelfinavir is a nonnucleoside class inhibitor against a range of HAdV types. Surprisingly, we found nelfinavir to inhibit HAdV infection, although nelfinavir was previously classified as being inactive against HAdV-C based on replication assays (105). It is the off-patent FDA-approved active pharmaceutical ingredient in Viracept. Nelfinavir was originally developed as an inhibitor against HIV aspartyl protease. It is orally bioavailable, with an inhibitory concentration in the low-nanomolar range (102, 104). Nelfinavir inhibits the replication of enveloped viruses, including severe acute respiratory syndrome coronavirus (SARS-CoV) (113), hepatitis C virus (114), as well as alpha-, beta-, and gammaherpesviruses (105). In the case of the alphaherpesvirus HSV-1, nelfinavir inhibits the envelopment of the capsid with cytoplasmic membranes. This coincided with the impaired glycosylation of gB and gC in the *trans*-Golgi network (TGN) (105, 111, 112, 115). Nelfinavir was reported to inhibit the activity of regulatory proteases in the Golgi compartment and the growth of cancer cells and to induce a wealth of other effects, including autophagy, endoplasmic reticulum (ER) stress, the unfolded protein response, and apoptosis (116–125; reviewed in references 126–128). It remains unknown if nelfinavir exerts these pleiotropic effects by interfering with diverse processes or a particular one.

Here, we demonstrate that nelfinavir inhibits the egress of HAdV particles without perturbing other viral replication steps, including entry, assembly, and maturation. Morphometric analyses of fluorescent plaques indicated that HAdV-C propagates by two distinct mechanisms, lytic and nonlytic. Lytic transmission led to comet-shaped convection-driven plaques, whereas nonlytic transmission gave rise to symmetric round plaques. Nelfinavir specifically suppressed the lytic spread of HAdVs, most prominently the HAdV-C types and -B14, but not other HAdVs such as A31 or D37. Incidentally, HAdV-C and -B14 replicate to considerable levels in Syrian hamsters, whereas other HAdV types do not (31, 129, 130). We infer that lytic infection could be a pathogenicity driver, at least in the hamster model.

The molecular mechanisms underlying cell lysis in AdV infection are not well understood, largely due to the lack of specific assays and inhibitors. Single-cell analyses combined with machine learning have started to identify specific features of lytic cells, such as increased intranuclear pressure compared to nonlytic cells (131). Lysis induced by HAdV was suggested to involve caspase-dependent functions and necrosis-like features (99, 132, 133). The best-characterized factor in HAdV cell lysis is ADP, a small membrane protein encoded by HAdV-C (90, 91, 134). ADP deletion mutants show a delayed onset of plaque formation (73, 107). Lysis is enhanced by increased ADP levels and tuned by posttranslational ADP processing (73, 74, 107). ADP has a single signal/anchor sequence, and its luminal domain is N- and O-glycosylated. The N-terminal segment is cleaved off in the Golgi lumen, and membrane-anchored ADP localizes to the inner nuclear membrane (92, 107, 108). Interestingly, two cysteine residues in the cytoplasmic domain adjacent to the transmembrane segment are palmitoylated (108, 135). S-palmitoylation is known to support the anchorage and sorting of host and viral membrane proteins. Accordingly, S-palmitoylation in the Golgi compartment facilitates protein oligomerization and virion assembly and entry, as shown for structural proteins of enveloped viruses, including SARS-CoV-1 S, vesicular stomatitis virus G, Sindbis virus E2, influenza virus hemagglutinin (HA), respiratory syncytial virus F, or rubella virus E1 and E2, as well as viroporin-mediated membrane permeabilization, including mouse hepatitis virus E protein, SARS-CoV-1 E protein, and Sindbis virus 6K (for reviews, see references 136 and 137).

Conspicuously, the cell lysis-defective HAdV mutant pm734.4 encodes a C2 mutant ADP with two point mutations in the transmembrane domain, C53R and M56L (108). The mutant ADP localizes to the ER and the nuclear envelope, but not the Golgi compartment, unlike the parental wild-type (wt) virus rec700. The localization of pm734.4 ADP is akin to the localization of HAdV-C2 ADP in nelfinavir-treated cells, which resist lysis and lack ADP localization in the Golgi compartment. We speculate that the palmitoylation of ADP in the Golgi compartment is crucial for ADP to enhance the rupture of the nuclear membrane in lytic HAdV-C egress. Nelfinavir may interfere with ADP palmitoylation either by inhibiting a palmitoyl-acyltransferase or by dispersing the donor substrate for protein palmitoylation, palmitoyl-coenzyme A (137). Remarkably, nelfinavir has a high log*P* value of 4.1 to 4.68 (138, 139) and partitions into lipophilic domains of the cell, including membranes. This is akin to another lipophilic drug with pleiotropic effects, the antiviral and anthelminthic compound niclosamide, which is a weak acid that acts as a protonophore extracting protons from acidic organelles and thereby inhibits virus entry and uncouples mitochondrial proton gradients (140, 141).

We noticed that ADP is not the sole lysis factor of HAdV. HAdV types lacking ADP, such as B types, also release their progeny by lysis albeit with efficacies that vary depending on the cell type (142–144). This is in agreement with the observation that HAdV types of the A, B, and D species form comet-shaped plaques and that ADP-deleted HAdV-C2 lyses the host cell and forms comet-shaped plaques albeit delayed and with lower efficacy than ADP-containing rec700 or HAdV-C2-dE3B-GFP. Conspicuously, other AdV proteins besides ADP were reported to interfere with cell lysis, such as the early region 4 open reading frame 4 (ORF4) protein, which induces nuclear envelope blebbing and promotes the loss of nuclear integrity (145, 146). This, together with diverse cellular mechanisms underlying force generation and membrane rupture, could compensate for the lack of ADP in some forms of lytic virus egress (51, 55, 146). We consider it unlikely that genetic variability of the inoculum accounts for the presence of lytic and nonlytic pathways since the inoculum was derived from an infectious DNA clone of HAdV-C2-dE3B-GFP and lacked any mutations affecting amino acid coding across many passages (72).

In addition to providing a new inhibitor of lytic HAdV propagation, nelfinavir revealed an alternative nonlytic HAdV transmission pathway that gives rise to slow-growing symmetrical plaques. This nonlytic pathway exists in unperturbed cells but is camouflaged by rapid and far-reaching lytic infection. The nonlytic egress pathway is likely a deterministic process. It is stable for at least 8 days (Fig. 5C). It remains to be explored if cells can switch between the lytic and the nonlytic pathways. Regardless, nonlytic egress from the nucleus bypasses the nuclear envelope and the plasma membrane. We speculate that the nonlytic pathway involves the sorting of HAdV particles to membrane sites where outward budding and scission occur. HAdV budding through the nuclear envelope could involve the WASH complex, akin to the nuclear release of large RNPs in Drosophila melanogaster and perhaps similar to HSV budding (147, 148). Cytoplasmic membrane budding could be enhanced by the ESCRT complex, which is known to release enveloped viruses such as HIV and also facultative-enveloped viruses such as hepatitis A virus (149–151). Alternatively, autophagy could sequester virions from the nucleus and upon fusion with the plasma membrane release virions from infected cells.

In conclusion, our work opens new therapeutic options for treating adenovirus disease, including acute and persistent infections. For example, HAdV-C persists in lymphocytes, which resist lytic infection, but also in epithelial cell lines under the repression of interferon and activation of the unfolded protein response sensor IRE-1a (152–157). Nelfinavir might be considered for anti-HAdV therapy, for example, prophylactically in hematopoietic stem cell recipients whose lives are threatened by the reactivation of HAdV-C (5, 6, 158).

## MATERIALS AND METHODS

### Viruses.

HAdV-C2-dE3B-GFP was previously described (72) (GenBank accession number MT277585). The virus was generated by the exchange of the viral E3b genome region with a reporter cassette harboring enhanced green fluorescent protein (GFP) under the control of a constitutively active cytomegalovirus (CMV) promoter. It was grown in A549 cells and purified by double-CsCl-gradient centrifugation (159). Aliquots supplemented with 10% (vol/vol) glycerol were stored at −80°C. HAdV-C2-dE3B-GFP was found to be homogeneous by SDS-PAGE and negative-stain analyses by transmission electron microscopy (EM). Recombinant HAdV-C2-dE3B-GFP-dADP was generated using homologous recombination according to Warming recombineering protocols (160, 161). For a detailed protocol, see the supplemental material. HAdV-C2-dE3B-GFP-dADP was plaque purified and amplified, followed by two rounds of CsCl purification (162). Aliquots containing 10% (vol/vol) glycerol were stored at −80°C. HAdV-C2-dE3B-GFP-dADP was found to be homogeneous by SDS-PAGE and negative-stain analyses by transmission EM. The lack of ADP expression was confirmed by Western immunostaining using the rabbit anti-HAdV-C2-ADP_78–93_ antibody, obtained from William S. M. Wold and Ann E. Tollefson (St. Louis University, St. Louis, MO, USA) (108).

HAdV types A31, B7, B11, B14a, B16, B34, C1, C6, D8, D30, and D37 were kindly provided by the late Thomas Adrian (Hannover Medical School, Germany) and were verified by DNA restriction analysis (163, 164). HAdV types B14 (19, 20) and B21a, isolate LRTI-6 (165), were kindly provided by Albert Heim (Hannover Medical School, Germany). HAdV-B3-pIX-FS2A-GFP and B35-pIX-FS2A-GFP contain an enhanced GFP open reading frame (ORF) genetically fused to the downstream end of the HAdV pIX gene using an autocleavage FS2A sequence (166–168). rec700 (169) and dl712 (170) were obtained from William S. M. Wold (St. Louis University, St. Louis, MO, USA). rec700 is a recombinant HAdV-C5 containing C2 sequences from nucleotides −236 to 2437 of the E3 transcription unit and comprises the C2 E3a ORFs 12.5K, 6.7K, 19K, and ADP as well as major parts of the E3b ORF RIDα (10.4K protein) (171). Mouse adenovirus 1 pIX-FS2A-GFP (MAdV-1-pIX-FS2A-GFP) and MAdV-3-pIX-FS2A-GFP were constructed as described previously (172, 173). HAdV-C2 and -C5 were obtained from Maarit Suomalainen (University of Zurich, Switzerland). HSV-1-CMV-GFP is a recombinant HSV-1 SC16 strain containing a CMV enhancer/promoter-driven enhanced GFP expression cassette in the US5 (gJ) locus (174) and was kindly provided by Cornel Fraefel (University of Zurich, Switzerland). HSV-1-CMV-GFP was propagated in Vero cells and purified by sucrose sedimentation as described previously (175, 176). All viruses were stored in small aliquots containing 10% (vol/vol) glycerol at −80°C.

### Cell lines.

A549 (human adenocarcinomic alveolar basal epithelium; ATCC CCL-185) cells, HeLa (human epithelial cervix carcinoma; ATCC CCL-2) cells, and HBECs (HBEC3-KT, normal human bronchial epithelium; ATCC CRL-4051) were obtained from the American Type Culture Collection (ATCC) (Manassas, VA, USA). HCE (normal human corneal epithelium) cells were obtained from Karl Matter (University College London, UK). CMT93 (mouse rectum carcinoma) cells were obtained from Susan Compton, Yale School of Medicine. A549, HeLa, HCE, and CMT-93 cell cultures were maintained in high-glucose Dulbecco’s modified Eagle’s medium (DMEM) (Thermo Fisher Scientific, Waltham, MA, USA) containing 7.5% (vol/vol) fetal calf serum (FCS) (Invitrogen, Carlsbad, CA, USA), 1% (vol/vol) l-glutamine (Sigma-Aldrich, St. Louis, MO, USA), and 1% (vol/vol) penicillin-streptomycin (Sigma-Aldrich, St. Louis, MO, USA) and subcultured following phosphate-buffered saline (PBS) washing and trypsinization (trypsin-EDTA; Sigma-Aldrich, St. Louis, MO, USA) biweekly. HBECs were maintained in endothelial-basal medium (ATCC, Manassas, VA, USA) and passaged 1:1 weekly following PBS washing and trypsinization. Cell cultures were grown under standard conditions (37°C, 5% CO_2_, and 95% humidity), and the passage number was limited to 20. The respective supplemented medium is referred to as supplemented medium.

### Compounds.

Nelfinavir mesylate (CAS number 159989-65-8) powder was obtained from MedChemExpress LLC (Monmouth Junction, NJ, USA) and Selleck Chemicals (Houston, TX, USA). The compound was dissolved in dimethyl sulfoxide (DMSO) (Sigma-Aldrich, St. Louis, MO, USA) at 100 mM and kept at −80°C or −20°C for long-term or working storage, respectively.

### Cellular impedance measurement.

Impedance-based assays were performed using the xCELLigence system (Roche Applied Science and ACEA Biosciences) as described previously (152, 153), according to the manufacturer’s instructions (177), in a cell culture environment (37°C, 5% CO_2_, and 95% humidity) in duplicates. The 16-well E plates have a gold-plated sensor array embedded in their glass bottom by which the electrical impedance across each well bottom is measured. The impedance per well, termed the cell index (CI), is recorded as a dimensionless quantity. The background CI was assessed following the addition of 50 μl supplemented medium to each well and equilibration in the incubation environment. After 30 min of equilibration, 9,000 A549 cells in 50 μl supplemented medium were added per well, and measurement was started.

For the quantification of nelfinavir toxicity, 50 μl of the supernatant was removed 18 h later and replaced with 2-fold-concentrated nelfinavir or DMSO solvent as the control dilution in supplemented medium (final nelfinavir concentration of 0.4 to 100 μM in 100 μl/well). The control was supplemented medium. Impedance was recorded every 15 min over 5 days. The TC_50_ indicates the concentration of nelfinavir that caused a 50% impedance reduction compared to the solvent-treated cells. The TC_50_ was calculated by nonlinear regression of the solvent-normalized CI over the concentration of nelfinavir.

For the quantification of nelfinavir effects on the cytopathogenicity of HAdV-C2-dE3B-GFP compared to HAdV-C2-dE3B-GFP-dADP infection, 50 μl of the supernatant was removed 18 h later and replaced with nelfinavir- and virus-supplemented medium. Twenty-five microliters of 4-fold-concentrated nelfinavir (final concentration, 0.4 to 100 μM) or the corresponding DMSO solvent control dilution (final concentration, 1%) in supplemented medium or supplemented medium only was added to 50 μl of medium containing cells. Additionally, 25 μl of a 4-fold-concentrated virus stock dilution was added (final inoculum, 1.68 × 10^6^ viral particles [VP]/well of HAdV-C2-dE3B-GFP and 2.68 × 10^6^ VP/well of HAdV-C2-dE3B-dADP, corresponding to ∼30 PFU/well). The delay of infection-induced cytotoxicity was calculated as the time point at which the CI of the infected cells had decreased by 50% relative to its maximum. Data analysis was performed using GraphPad (version 8.1.2; GraphPad Software, Inc.), and curve fitting was performed using three-parameter inhibitor concentration-versus-response nonlinear regression.

### Fluorescence-based plaque-forming assay.

Per 96-well plate, 15,000 A549 cells, 10,000 HeLa cells, 30,000 HBECs, 30,000 HCE cells, or 30,000 CMT-93 cells were seeded in 100 μl of the respective supplemented medium and allowed to settle for 1 h at room temperature (RT) prior to cell culture incubation at 37°C with 5% CO_2_ and 95% humidity. The following day, the medium was replaced with 50 μl of the respective virus stock dilution giving rise to 5 to 50 plaques per 96-well plate. Fifty microliters of nelfinavir to obtain a 0.1 to 50 μM final concentration or the DMSO solvent control was also added, both in supplemented medium. For each experiment, a noninfected, treated control was performed. For uphill plaque assays, the medium volume was increased to 150 μl with identical virus and drug concentrations. For wash-in/washout experiments, the virus was incubated on cells in supplemented medium for 1 h at 37°C, cells were washed with PBS, and a 100-μl drug dilution in supplemented medium was added. All experiments were performed in four technical replicates or as indicated. Cells were incubated under standard cell culture conditions. At the indicated times postinfection, the cells were fixed, and the nuclei were stained for 1 h at RT by the addition of 33 μl 16% (wt/vol) paraformaldehyde (PFA) and 4 μg/ml Hoechst 33342 (Sigma-Aldrich, St. Louis, MO, USA) in PBS. Cells were washed three times with PBS and stored in PBS supplemented with 0.02% N_3_ for infections with viruses harboring a GFP transgene. For wild-type (wt) viruses, cells were quenched in PBS supplemented with 50 mM NH_4_Cl, permeabilized using 0.2% (vol/vol) Triton X-100 in PBS, and blocked with 0.5% (wt/vol) bovine serum albumin (BSA) in PBS. Cells were incubated with 381.7 ng/ml mouse anti-HAdV hexon protein antibody (Mab8052; Sigma-Aldrich, St. Louis, MO, USA) and subsequently stained using 2 μg/ml goat anti-mouse-Alexa Fluor 594 (catalog number A21203 or A32742; Thermo Fisher Scientific, Waltham, MA, USA). Plates were imaged on either an IXM-XL or an IXM-C automated high-throughput fluorescence microscope (Molecular Devices, San Jose, CA, USA) using a 4× objective in wide-field mode. Hoechst staining was recorded in the 4′,6-diamidino-2-phenylindole (DAPI) channel, the fluorescein isothiocyanate (FITC)/GFP channel was acquired for viral GFP, and the tetramethyl rhodamine isothiocyanate (TRITC)/Texas Red channel was acquired for hexon immunofluorescence staining.

### Therapeutic index measurement.

The infection phenotype for each well was quantified using Plaque2.0 (101). The number of plaques was determined based on the infection signal (viral GFP or hexon immunofluorescence staining). Nuclei stained with Hoechst were segmented by using CellProfiler (178). Infected nuclei were classified based on the median infection signal per nucleus in CellProfiler. Data were plotted, and the EC_50_ (infected and treated cells), the TC_50_ (noninfected, treated cells), as well as the corresponding standard errors (SE) were determined using curve fitting in GraphPad (version 8.1.2; GraphPad Software, Inc.) using three-parameter inhibitor concentration-versus-response nonlinear regression. The mean TI_50_ was calculated as the EC_50_/TC_50_ ratio of the means. The TI_50_ SE was calculated by error propagation.

### Quantification of viral protein expression.

Infection, HAdV hexon immunofluorescence staining, and imaging were performed in technical quadruplicates, as described above for the microscopic plaque assay. Single nuclei (Hoechst) were segmented using CellProfiler (178). Median GFP and hexon signals per nucleus were measured, and infected nuclei were classified using the median GFP or hexon signal per nucleus. Subsequently, the mean and standard deviation (SD) over all infected nuclei per well were calculated using R version 3.3.2 (179). Data were plotted using GraphPad (version 8.1.2; GraphPad Software, Inc.).

### Transmission electron microscopy.

A549 cells grown on alcian blue-treated coverslips were infected with HAdV-C2-dE3B-GFP in medium supplemented with 0, 1.25, or 3 μM nelfinavir and cultured for 40 h under standard cell culture conditions. The samples were washed with ice-cold 0.1 M cacodylate buffer (pH 7.4) and fixed at 4°C in 0.1 M ice-cold cacodylate buffer (pH 7.4) supplemented with 2.5% (vol/vol) glutaraldehyde and 0.5 mg/ml ruthenium red for 1 h. Cells were washed with 0.1 M cacodylate buffer (pH 7.4) and postfixed at RT in 0.05 M cacodylate buffer (pH 7.4) supplemented with 0.5% (vol/vol) OsO_4_ and 0.25 mg/ml ruthenium red for 1 h. Following washing with 0.1 M cacodylate buffer (pH 7.36) and H_2_O, the samples were incubated in 2% (vol/vol) uranyl acetate at 4°C overnight. The samples were dehydrated in acetone and embedded in Epon as described previously (180). Slices of 85 nm were obtained (Ultracut UCT; Leica, Wetzlar, Germany) and stained with uranyl acetate.

### HAdV-C5 production in the presence of nelfinavir.

HAdV-C5 was amplified in medium containing 0, 1.25, or 3 μM nelfinavir for 4 days. Cells were harvested and disrupted by three freeze-thaw cycles. The cell debris was removed by freon extraction, and mature full HAdV virions were purified by two rounds of CsCl gradient ultracentrifugation (162). The protein concentration was determined by a bicinchoninic acid (BCA) assay (Pierce BCA protein assay kit; Thermo Fisher Scientific, Waltham, MA, USA). For long-term storage, virus stocks were supplemented with 10% (vol/vol) glycerol and kept at −80°C.

### Negative-staining electron microscopy.

Double-CsCl-gradient-purified HAdV particles were adhered to collodion and 2% (vol/vol) amyl acetate film-covered grids (300-mesh Formvar/carbon-supported copper support films; Electron Microscopy Sciences, Hatfield, PA, USA). Viral particles were negatively stained with 2% (vol/vol) uranyl acetate and viewed on a transmission electron microscope (CM100; Philips, Amsterdam, Netherlands) at 100 kV. Images were acquired using a charge-coupled-device (CCD) camera (Orius SC1000 with 4,000 by 2,600 pixels [px]; Gatan, Pleasanton, CA, USA).

### Western blot analysis of HAdV protease activity.

Double-CsCl-purified HAdV particles grown in the presence or absence of nelfinavir (HAdV-C5^±Nelfinavir^ stocks) and a size standard (PageRuler plus; Thermo Fisher Scientific, Waltham, MA, USA) were size separated on a 12% acrylamide gel under reducing conditions and transferred to a polyvinylidene difluoride (PVDF) membrane. HAdV proteins were detected using primary antibodies, 1:10,000 R72 rabbit antifiber (181), 1:1,000 rabbit anti-pVI/VI (51), and 1:1,000 R3 rabbit anti-pVII/VII (Ulf Pettersson, Uppsala University), and visualized using goat anti-rabbit-horseradish peroxidase (HRP) (catalog number 7074; Cell Signaling Technology, Danvers, MA, USA) and the ECL Prime Western blotting detection reagent (GE Healthcare, Pittsburgh, PA, USA). The membranes were luminescence imaged on an Amersham 680 imager (GE Healthcare, Pittsburgh, PA, USA).

### Determination of nuclear size.

Infection and nelfinavir treatment of A549 cells were performed as described above for the microscopic plaque assay with a cell seeding density of 15,000 cells/well. Wells were imaged with an IXM-C automated high-throughput fluorescence microscope (Molecular Devices, San Jose, CA, USA) using a 40× objective (numerical aperture [NA], 0.95) in confocal mode (62-μm pinhole). The DAPI channel was acquired for nuclear Hoechst staining, the FITC/GFP channel was acquired for viral GFP, the TRITC/Texas Red channel was acquired for immunofluorescence ADP staining, and the Cy5 channel was acquired for the *N*-hydroxysuccinimide (NHS)-ester signal. Thirty z steps with a 0.5-μm step size were acquired for each channel, and maximal projections were calculated. Image analysis was performed using CellProfiler (178). Nucleus areas were segmented based on the thresholded Hoechst signal. Infected cells were classified based on a fixed threshold for the median nuclear GFP intensity. Data processing was performed in R version 3.3.2 (179). Statistical analysis was performed in GraphPad (version 8.1.2; GraphPad Software, Inc.) using the nonparametric Kolmogorov-Smirnov test.

### Cell binding assay of virus.

A549 cells were seeded at 7,500 cells per 96-well in full DMEM and allowed to attach overnight under standard cell culture conditions. The next day, the medium was replaced with 3 × 10^8^ VP/well of double-CsCl-purified HAdV-C5^±Nelfinavir^ stocks in 100 μl ice-cold supplemented medium and kept on ice for 30 min. Following a 15-min entry phase under standard cell culture conditions, the cells were fixed, and the nuclei were stained for 1 h at RT by the addition of 33 μl 16% PFA and 4 μg/ml Hoechst 33342 (Sigma-Aldrich, St. Louis, MO, USA) in PBS. Following the above-described immunofluorescence staining procedure, the cell-bound HAdV virions were stained using 9C12 mouse antihexon (developed by Laurence Fayadat and Wiebe Olijve, obtained from the Developmental Studies Hybridoma Bank developed under the auspices of the National Institute of Child Health and Human Development and maintained by the University of Iowa, Iowa City, IA, USA) (182) and goat anti-mouse-Alexa Fluor 488 (catalog number A11029; Thermo Fisher Scientific, Waltham, MA, USA). The total area was identified by Alexa Fluor 647 NHS-ester staining (catalog number A20006; Thermo Fisher Scientific, Waltham, MA, USA). Maximal projections of confocal z-stacks (25 z steps spaced 1 μm apart) were acquired on an SP5 resonant Avalanche photodiode (APD) (Leica, Wetzlar, Germany) at 1.7× zoom using a 63× glycerol objective (numerical aperture, 1.4).

### Assessment of HAdV infectivity of HAdV-C5^±Nelfinavir^.

Fifteen thousand A549 cells were seeded per 96-well in full DMEM and allowed to attach overnight under standard cell culture conditions. The next day, the medium was replaced by double-CsCl-purified HAdV-C5^±Nelfinavir^ stocks at 50 to 0.001 pg/well of a BCA-based viral protein and incubated under standard cell culture conditions. Cells were fixed at 52 hpi, stained for HAdV hexon expression, and imaged according to the procedure described above for the image-based plaque assay. Images were quantified using Plaque2.0 (101). Nuclei were segmented based on the Hoechst signal. Infected cells were segmented based on the hexon immunofluorescence staining signal.

### Egress assay.

A549 cells were seeded at 480,000 cells per 6-well plate in full DMEM and infected at 1,100 PFU HAdV-C2-dE3B-GFP per well the next day. Following 1 h of warm incubation, the supernatant was removed, and cells were washed with PBS and detached by trypsin digestion. Infected cells were centrifuged and resuspended in fresh medium to remove any unbound input virus and seeded at 180,000 cells/12-well plate in medium supplemented with 1.25, 3, or 10 μM nelfinavir or equivalent amounts of the DMSO solvent control. At the indicated times postinfection, the supernatant was harvested and cleared by centrifugation. Two hundred microliters of PBS/well was added to the infected monolayer. Cells were disrupted by three freeze-thaw cycles, and freon extraction was performed. The supernatant and cell lysate were stored at 4°C until titration on naive A549 cells. PFA-fixed, Hoechst-stained cells were imaged at 44 hpi using a 4× objective (NA, 0.20) on an IXM-XL epifluorescence microscope (Molecular Devices, San Jose, CA, USA). GFP-positive infected cells were classified based on the median nuclear GFP intensity using automated image analysis by CellProfiler (178).

### Quantification of infectious progeny production.

Four hundred eighty thousand A549 cells were seeded per 6-well dish, inoculated with 1,100 PFU HAdV-C2-dE3B-GFP/well for 1 h at 37°C, washed with PBS, and detached by trypsin digestion. Infected cells were centrifuged and resuspended in fresh medium to remove any unbound input virus. Cells were seeded at 180,000 cells/12-well plate in medium supplemented with 1.25, 3, or 10 μM nelfinavir or the respective DMSO solvent control. Viral progeny in the cell monolayer and supernatant was harvested at the indicated times postinfection by three freeze-thaw cycles. The lysates were cleared by centrifugation and stored at 4°C until titration on naive A549 cells. PFA-fixed, Hoechst-stained cells were imaged at 44 hpi using a 4× objective on an IXM-XL epifluorescence microscope (Molecular Devices, San Jose, CA, USA). GFP-positive infected cells were classified based on the median nuclear GFP intensity using automated image analysis by CellProfiler (178). The yield per 12-well plate was extrapolated by linear regression of the number of infected cells per microliter of the harvested whole-well lysate using GraphPad (version 8.1.2; GraphPad Software, Inc.).

### Quantification of the antiviral potency of nelfinavir.

Infection was performed as described above for the microscopic plaque assay. Cells were incubated with an inoculum ranging between 10 and 2,560 PFU/well HAdV-C2-dE3B-GFP for 1 h at 37°C. Cells were washed with PBS and 100 μl phenol-free DMEM (Thermo Fisher Scientific, Waltham, MA, USA) supplemented with 1% penicillin-streptomycin (Sigma-Aldrich, St. Louis, MO, USA), 1% l-glutamine (Sigma-Aldrich, St. Louis, MO, USA), 7.5% fetal bovine serum (FBS) (Invitrogen, Carlsbad, CA, USA), 1% nonessential amino acids (Sigma-Aldrich, St. Louis, MO, USA), 1% 100 mM sodium pyruvate (Thermo Fisher Scientific, Waltham, MA, USA), 0.25 ng/ml Hoechst 33342 (Sigma-Aldrich, St. Louis, MO, USA), and 1 μg/ml propidium iodide (PI) (Molecular Probes, Eugene, OR, USA). Plates were imaged at the indicated times postinfection on an IXM-C automated high-throughput fluorescence microscope (Molecular Devices, San Jose, CA, USA) using a 40× objective (NA, 0.95) in confocal mode (62-μm pinhole). The DAPI channel was acquired for nuclear Hoechst staining, the FITC/GFP channel was acquired for viral GFP, and the Cy5 channel was acquired for the PI signal. Thirty z steps with a 0.5-μm step size were acquired for each channel, and maximal projections were calculated.

### Morphological plaque characterization.

Plaques were segmented in Plaque2.0 (101), and plaque region eccentricity was measured as a fraction of the distance between the two focal points of the ellipse divided by the length of the major axis. Only plaque regions consisting of at least five infected cells (≥6,000 px^2^) with a centroid located 600 px from the well rim were considered to exclude spatial limitations. Plaque roundness was calculated as 1 − eccentricity, as follows: roundness = 1 − (4π × area)/(perimeter^2^).

Statistical analysis was performed in GraphPad (version 8.1.2; GraphPad Software, Inc.) using the nonparametric Kolmogorov-Smirnov test.

### Confocal microscopy of ADP localization.

Infection and immunofluorescence stainings were performed as described above for the microscopic plaque assay with a cell seeding density of 3,000 cells/well. Cells were incubated with a 1:1,000 dilution of rabbit anti-HAdV-C2-ADP_87–101_ antibody (108) and subsequently stained using donkey anti-rabbit-Alexa Fluor 594 (catalog number A21207; Thermo Fisher Scientific, Waltham, MA, USA) and 0.2 μg/ml NHS-ester (Life Technologies, Carlsbad, CA, USA) for whole-cell outlines. Plates were imaged on an IXM-C automated high-throughput fluorescence microscope (Molecular Devices, San Jose, CA, USA) using a 40× objective (NA, 0.95) in confocal mode (62-μm pinhole). The DAPI channel was acquired for nuclear Hoechst staining, the FITC/GFP channel was acquired for viral GFP, the TRITC/Texas Red channel was acquired for immunofluorescence ADP staining, and the Cy5 channel was acquired for the NHS-ester signal. Thirty z steps with a 0.5-μm step size were acquired for each channel, and maximal projections were calculated. Image analysis was performed using CellProfiler (178). Nuclei and whole-cell areas were segmented based on thresholded Hoechst and NHS-ester signals, respectively. The nuclear rim was defined as a 10-pixel-wide area around the nuclear border. Infected cells were classified based on the whole-cell 5%-quantile GFP intensity. Whole-cell and nuclear rim mean TRITC/Texas Red (detecting ADP) intensities as well as whole-cell 5-pixel granularity per infected cell were normalized by the mean of the measurement over all infected cells of the solvent control. Data processing was performed in R version 3.3.2 (179). Statistical analysis was performed in GraphPad (version 8.1.2; GraphPad Software, Inc.) using the nonparametric Kolmogorov-Smirnov test.

### Western blot analysis of ADP processing.

Four hundred eighty thousand A549 cells were seeded per 6-well plate, incubated overnight, and inoculated with HAdV-C2-dE3B-GFP at 22,000 PFU/well in 1.2 ml full DMEM supplemented with 0 to 10 μM nelfinavir. Following 44 h of incubation in standard cell culture medium, cells were placed on ice, and the supernatant was removed. The cells were washed twice with ice-cold PBS. Cells were lysed in 100 μl COS lysis buffer (20 mM Tris-HCl [pH 7.4], 100 mM NaCl, 1 mM EDTA, 1% Triton X-100, 1 mM dithiothreitol [DTT], 25 mM β-glycerophosphate disodium, 25 mM NaF, 1 mM Na_3_VO_4_, 1× protease inhibitors [Mini complete; Roche, Basel, Switzerland]) for 5 min on ice. The supernatant and washing PBS were collected, and cells were pelleted by centrifugation at 16,000 × *g* for 5 min at 4°C. Lysates were scraped off and used to resuspend the pelleted cells. Following another centrifugation step, the supernatant was collected and stored at −20°C. Samples of 15 μl of the lysate were supplemented with SDS-containing loading buffer (0.35 M Tris-HCl [pH 6.8], 0.28% SDS, 30 g/liter DTT, 0.6 g/liter bromophenol blue). Samples were denatured at 95°C for 5 min, and proteins were separated on a denaturing 15% acrylamide gel. Proteins transferred to a PVDF membrane were detected with a 1:1,000 dilution of a rabbit anti-HAdV-C2-ADP_78–93_ antibody (108), followed by goat anti-rabbit-HRP (catalog number 7074; Cell Signaling Technology, Danvers, MA, USA). Protein bands were visualized using the ECL Prime Western blotting detection reagent (GE Healthcare, Pittsburgh, PA, USA), and luminescence was imaged on an Amersham 680 imager (GE Healthcare, Pittsburgh, PA, USA).

### Neutralization of HAdV cell-free progeny.

A549 cells were seeded at 15,000 cells per well of a 96-well plate, incubated overnight, and inoculated with HAdV-C2-dE3B-GFP at 34 PFU/well for 1 h at 37°C. Unbound inoculum was removed, and cells were washed with PBS, before treatment with 0.25 ng/ml Hoechst (Sigma-Aldrich, St. Louis, MO, USA)-supplemented DMEM containing a 1:12 dilution of HAdV-C2/5-neutralizing dog serum (kindly supplied by Anja Ehrhardt, University of Witten/Herdecke, Germany) (183) supplemented with 40% (vol/vol) glycerol, control goat serum (Thermo Fisher Scientific, Waltham, MA, USA) supplemented with 40% [vol/vol] glycerol, or the corresponding volume of glycerol only. Cells were imaged using a 4× objective (NA, 0.20) on an IXM-XL epifluorescence microscope (Molecular Devices, San Jose, CA, USA).

### Crystal violet-stained plaques.

Plaque shapes were also assessed by a conventional crystal violet-stained plaque assay performed on A549 cells in liquid-supplemented DMEM. All infections were performed at 37°C with 95% humidity and a 5% CO_2_ atmosphere. At the indicated times postinfection, cells were fixed and stained for 60 min with a PBS solution containing 3 mg/ml crystal violet and 4% PFA added directly to the medium from a 16% stock solution. Plates were destained in H_2_O, dried, and imaged using a standard 20-megapixel phone camera under white-light illumination.

## Supplementary Material

Supplemental file 1

Supplemental file 2

Supplemental file 3
