# Supplementary material for: The FDA-Approved Drug Nelfinavir Inhibits Lytic Cell-Free but Not Cell-Associated Nonlytic Transmission of Human Adenovirus
Source: Antimicrob Agents Chemother. 2020 Aug 20;64(9):e01002-20. doi: 10.1128/AAC.01002-20 (PMC7449217; doi:10.1128/AAC.01002-20)
Supplement: Supplemental file 1 [file AAC.01002-20-s0001.pdf]

Supplemental Material

Georgi F, Andriasyan V, Witte R, Murer L, Hemmi S, Yu L, Grove M, Meili N, Kuttler F, Yakimovich A, Turcatti G, Greber UF. 2020. The FDA-approved drug Nelfinavir inhibits lytic cell-free, but not cell-associated non-lytic transmission of human adenovirus.

Supplementary Figures

Supplementary Figure 1: Nelfinavir exhibits low toxicity.

A Cell index (CI) profiles of uninfected A549 shows little signs of toxicity up to 6.25 µM of Nelfinavir during more than 4 days of incubation. Impedance was recorded every 15 min using xCELLigence. Vertical dotted line shows the time of drug addition.

B Nelfinavir does not affect infection-induced nuclear swelling in HAdV-C2-dE3B-GFP infected A549 cells at 44 hpi. Each violin symbol represents the areas of 99 nuclei from four technical replicates. Kolmogorov-Smirnov test indicated p value ≤0.05 (*), and ≤0.0001 (****), or no significant differences. Solid red lines indicate median, dotted red lines the 5-95% quartile. Horizontal dotted grey line represents the median of untreated, uninfected nuclei. Note that 20 µM Nelfinavir induced the shrinkage of the nuclei in both infected and uninfected cells, indicative of high dosage toxicity.

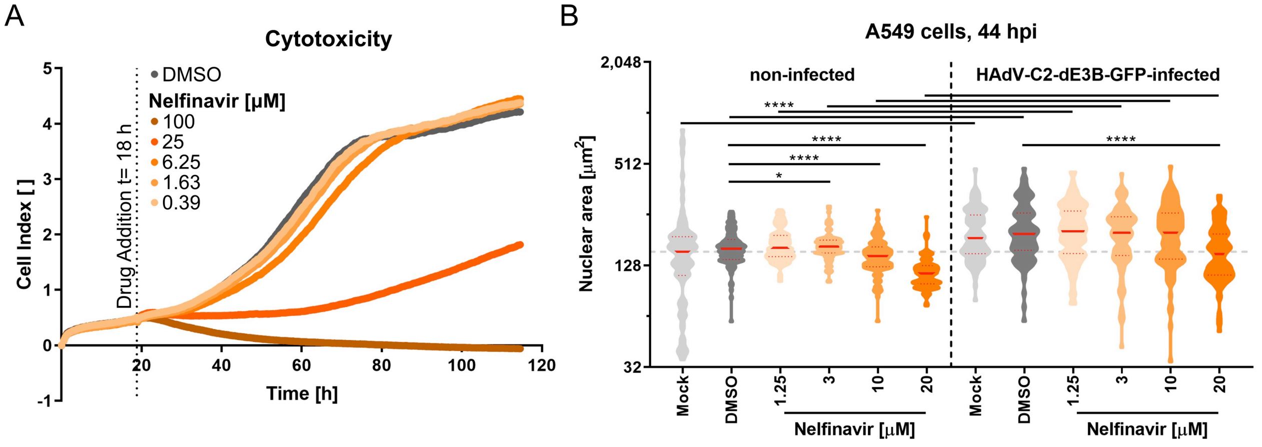

Supplementary Figure 2: Characterization of round plaque phenotypes revealed by Nelfinavir.

A Plate tilting to direct convection of medium fluids does not affect the spatio-temporal spreading pattern of HAdV-C2-dE3B-GFP in presence of Nelfinavir. Nelfinavir delays the formation of plaques in a concentration-dependent manner. Early plaques in absence of Nelfinavir show comet-shaped morphologies (red squares 1, 2), while late plaques in presence of Nelfinavir appear round (red square 3). GFP intensities are shown as 16-color LUT. White arrow indicates direction of elongation of comet plaques (uphill flow of convection). Scale bar is 1 mm.

B Topological views of plaque morphologies. Red numbers correspond to regions of interest indicated by red squares in panel **A** with equivalent x- and y-axes assignement. GFP expression along the z-axis. GFP intensity is shown as rainbow colour LUT, indicating the original of plaque formation where GFP intensity peaks (purple).

C Delay in plaque formation by Nelfinavir. Data points represent means of 12 replicates from two experiments, including the example well micrographs shown in panel **A**. Error bars indicate standard deviations. DMSO-treated infected wells are shown in green, infected wells treated with 1.25, 3 or 10 μM Nelfinavir are shown in shades of orange.

D Time-resolved analyses of plaque roundness compared to size in presence of Nelfinavir treatment (shades of orange). Plaques were scored if they contained least five infected cells (≥1,500 μm^2 indicated by the dotted vertical lines). Data points represent a single plaque, each located in a central region of a well, and were collected in two independent experiments. Representative micrographs are shown in panel **A**. Note that the plaque morphologies in control wells could not be quantified after 3 dpi, due to extensive virus dissemination.

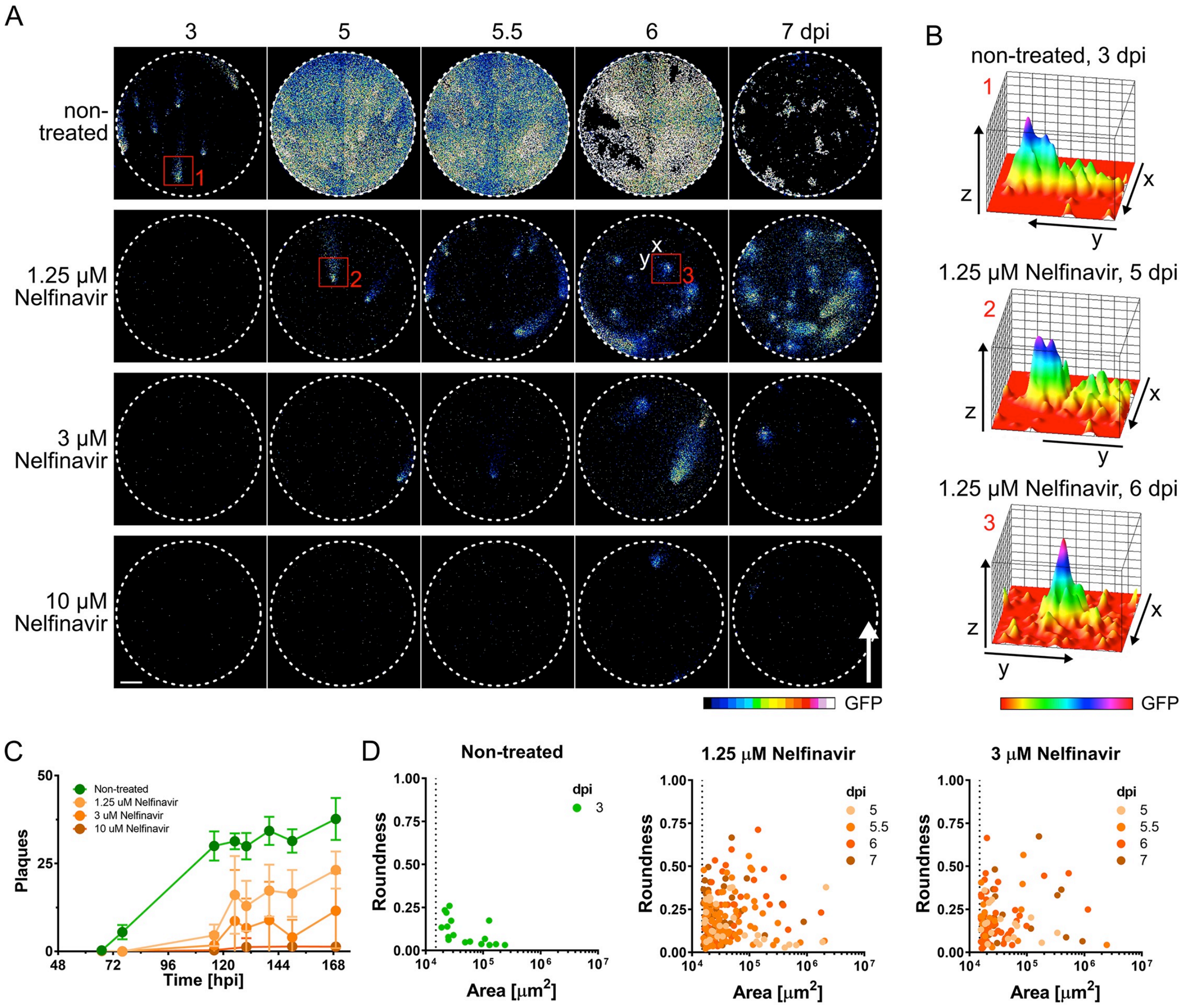
A
3
5
5.5
6
7 dpi
non-treated
1.25 µM Nelfinavir
3 µM Nelfinavir
10 µM Nelfinavir
1
2
3
x
y
GFP
B
non-treated, 3 dpi
1.25 µM Nelfinavir, 5 dpi
1.25 µM Nelfinavir, 6 dpi
z
x
y
GFP
C
Non-treated
1.25 uM Nelfinavir
3 uM Nelfinavir
10 uM Nelfinavir
Plaques
Time [hpi]
D
Non-treated
1.25 µM Nelfinavir
3 µM Nelfinavir
dpi
Roundness
Area [µm2]

Supplementary Figure 3: The mode of action of Nelfinavir is ADP-dependent irrespective of E3B deletion.

A Lack of ADP in HAdV-C2-dE3B-GFP-dADP infected cells 44 hpi shown by indirect immunofluorescence. HAdV-C2-dE3B-GFP-dADP infected cells compared to dl712, and the ADP-deleted HAdV-C5/2 rec700 mutant. Nuclei are shown in blue, GFP signal in green, ADP in red and cells in grey. Scale bar is 20 µm.

B Western blot analyses of HAdV-C2-dE3B-GFP-dADP compared to HAdV-C2-dE3B-GFP infected cells 48 and 72 hpi. Full length ADP runs at 19 kDa apparent mass, and the cleaved 16 kDa form at 14 kDa apparent mass.

C Negative stain EM micrographs of purified HAdV-C2-dE3B-GFP and HAdV-C2-dE3B-GFP-dADP particles. Scale bar 200 nm.

D, E Mean cell index profiles from impedance measurements of infected A549 cells performed in two technical replicates. Nelfinavir inhibits cytotoxicity in HAdV-C2-dE3B-GFP infected cells (**C**, green profile), but not in cells infected with the ADP-deletion mutant (**D**, red profile). Impedance was recorded every 15 min using xCELLigence. Each time point represents the average value from two replicates with standard deviations. The time on the x-axis indicates hours after cell seeding. Vertical lines show the time of infection and drug addition. Profiles of non-infected cells are shown in blue. The concentrations of Nelfinavir are represented by different shades of orange.

F Plaque formation in ADP-deleted HAdV-C2-dE3B-GFP and dl712 compared to HAdV-C2-dE3B-GFP and rec700 in A549. Cells were infected with $1.1*10^5$ VP / well. Nuclei shown in blue, GFP in green and hexon staining in red. Scale bar 1 mm. Dotted line indicates well outline. Note that lack of ADP delays plaque formation by 1 day without affecting plaque shape.

G Therapeutic index (TI_{50}), based on images shown in panel **H** and three additional technical replicates for HAdV-C harbouring ADP (HAdV-C2-dE3B-GFP and rec700) compared to ADP-deleted HAdV-C (HAdV-C2-dE3B-GFP-dADP, dl712). Plaque quantification was based on hexon staining. For detailed information and statistics, see Supplementary Table 2.

H Representative images of hexon-stained HAdV-C infection of A549 cells treated with Nelfinavir (50 to 0.1 µM, from left to right, orange triangle). Cells were fixed at 96 hpi (HAdV-C2-dE3B-GFP and rec700) or 112 hpi (HAdV-C2-dE3B-GFP-dADP, dl712). Nuclei in blue, hexon in red. Scale bar 1 mm. Dotted line indicates well outline.

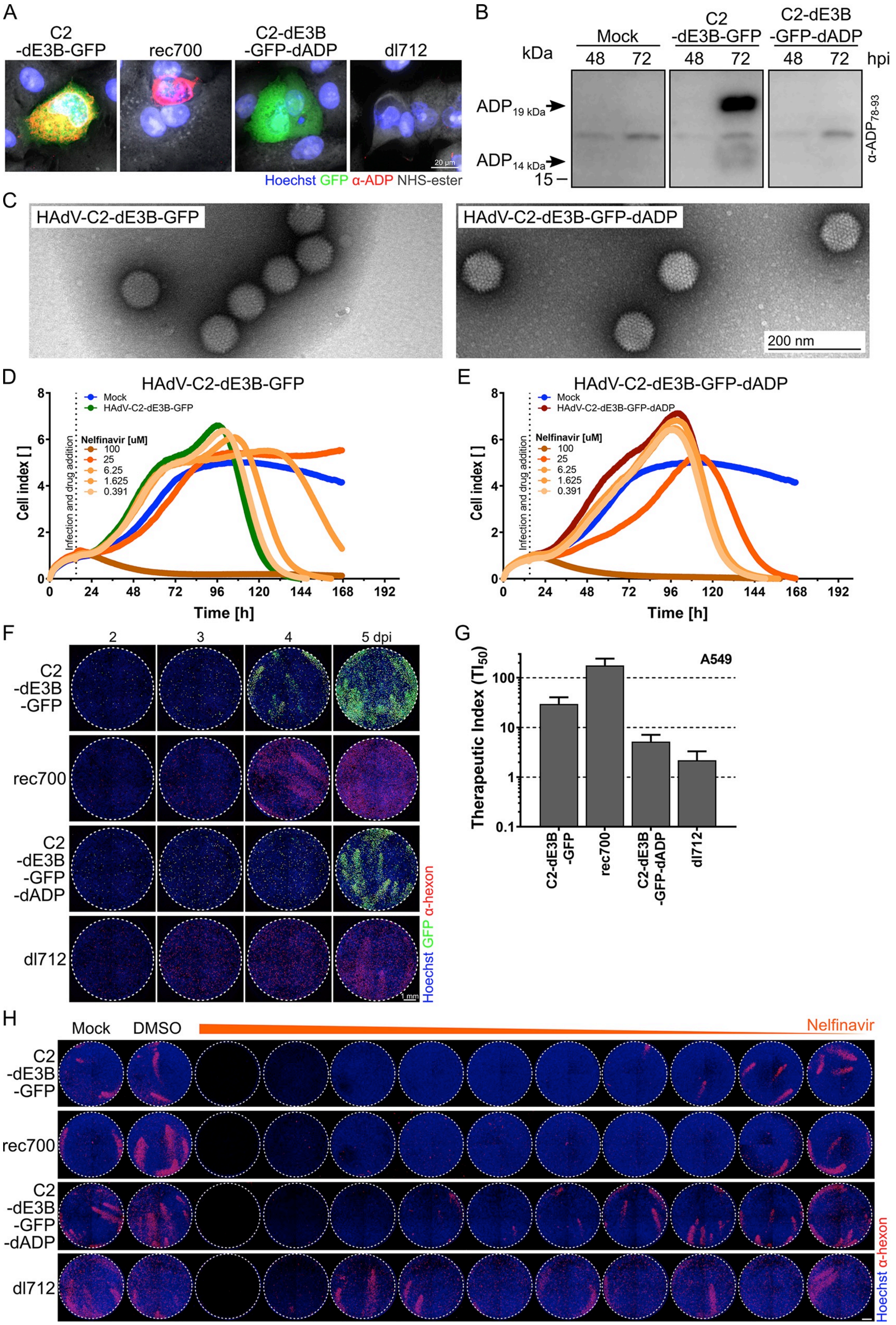
A
C2 -dE3B-GFP
rec700
C2-dE3B -GFP-dADP
dl712
20 µm
Hoechst GFP α-ADP NHS-ester
B
kDa
Mock
C2 -dE3B-GFP
C2-dE3B -GFP-dADP
48
72
hpi
ADP19 kDa
ADP14 kDa
15
α-ADP78-93
C
HAdV-C2-dE3B-GFP
HAdV-C2-dE3B-GFP-dADP
200 nm
D
HAdV-C2-dE3B-GFP
Mock
HAdV-C2-dE3B-GFP
Nelfinavir [uM]
100
25
6.25
1.625
0.391
Infection and drug addition
Cell index [ ]
Time [h]
E
HAdV-C2-dE3B-GFP-dADP
Mock
HAdV-C2-dE3B-GFP-dADP
F
2
3
4
5 dpi
C2 -dE3B -GFP
rec700
C2 -dE3B -GFP -dADP
dl712
Hoechst GFP α-hexon
1 mm
G
A549
Therapeutic Index (TI50)
C2-dE3B -GFP
rec700
C2-dE3B -GFP-dADP
dl712
H
Mock
DMSO
Nelfinavir
C2 -dE3B -GFP
rec700
C2 -dE3B -GFP -dADP
dl712
Hoechst α-hexon

Supplementary Tables

Supplementary Table 1: Summary of top hits identified in the HAdV AntiVir screen.

An accompanying manuscript (1) tested the Prestwick Chemical Library (PCL) for potentially repurposable inhibitors of HAdV infection. The data were acquired and analysed by two independent research teams at UZH and EPFL. The mean infection scores normalized to the negative control (neg. ctr.) obtained by both teams are listed for the four top hits. 3'-deoxy-3'-fluorothymidine (DFT) was used as positive control (pos. ctr.). Toxicity in absence of infection was tested as outlined in experimental procedures.

Supplementary Table 2: Therapeutic indices (TI_{50}) of Nelfinavir against AdVs in different cell lines.

Different cell lines were infected and fixed as indicated. Plaque numbers were quantified based on GFP expression the viral genome or hexon immunofluorescence. EC_{50} indicates the effective concentration reducing the plaque numbers by 50% per well. TC_{50} is the toxic concentration of Nelfinavir reducing the number of nuclei by 50% per well. TI_{50} is the ratio of TC_{50} to EC_{50}. Data are presented as means from indicated number of technical replicates, including the standard error (SE). Plaque numbers were quantified using Plaque2.0, and nuclei from Hoechst staining and CellProfiler analyses. Non-linear regression was performed in GraphPad.

Supplementary Methods

Generation of HAdV-C2-dE3B-GFP-dADP

As a first, a pKSB2-based bacterial artificial chromosome vector pKSB2-AdV-C2-dE3B_GFP carrying the genome of HAdV-C2-dE3B-GFP (2) was generated starting with pKSB2-AdV-C2-LARAzeo containing left and right terminal HAdV-C2 fragments. To generate pKSB2-AdV-C2-LARAzeo, two PCR-generated fragments were first cloned into pBluescript (pBl). The first fragment encompassed 853 bp of the HAdV-C2 left end sequence and was PCR-amplified using the forward primer 5'-ataagaatGCGGCCGCTAGGGATAACAGGGTAAT catcatcataatataccttattttgg-3' inserting the restriction sites NotI and I-SceI, and the reverse primer 5'- CTCTCTACTAGTAATAAGTCAATCCCTTCCTGC -3' inserting the restriction site SpeI. HAdV-C2-dE3B-GFP genomic DNA isolated from infected A549 cells was used as template. The NotI and SpeI restriction sites were used to clone the left arm fragment into pBl. The second fragment encompassed 853 bp of the HAdV-C2 right end sequence and was PCR-amplified using the forward primer 5'- agagagACTAGTaaaaacatttaaacattagaagcctg-3' adding the restriction sites SpeI and the reverse primer 5'- gcgcaagcttATTACCCTGTTATCCCTAcatcatcataatataccttattttgg-3' adding the sites I-SceI and HindIII. This fragment was cloned into pBl-AdV2-C2-LA by SpeI and HindIII restriction sites. A SpeI fragment containing the zeocine resistance marker from pcDNA3.1 zeo (Invitrogen) and generated by PCR using the forward 5'-GACTAGTTTTTCG GATCTGATCAGCACG-3' and reverse primer 5-GACTAGTGGAAAACGATT CCGAAGCCC-3' was cloned into the SpeI of pBl-AdV-C2-LARA, connecting the two HAdV-C2 arms and resulting in pBl-AdV-C2-LARAzeo. In order to transfer the AdV-C2-LARAzeo cassette to the BACmid pKSB2, the NotI-HindIII fragment containing this sequence was ligated with the NotI-HindIII-restricted pKSB2 vector. Colonies containing pKSB2-AdV-C2-LARAzeo were selected using chloramphenicol and zeocin at concentrations of 10 μg / ml and 25 μg / ml, respectively. In order to generate pKSB2-AdV-C2-dE3B_GFP, homologous recombination was performed in SW102 bacteria using AatII and ApaLI-restricted pKSB2-AdV-C2-LARAzeo and HAdV-C2-dE3B-GFP genomic DNA isolated from infected A549 cells.

HAdV-C2-dE3B-GFP-dADP was generated using two recombineering steps. In a first, the galK cassette was introduced into pKSB2-AdV-C2_dE3B_GFP to replace the ADP sequence. The GalK cassette was amplified using the forward primer 5'- ACTGCAAATTTGATCAAACC CAGCTTCAGCTTGCCTGCTCCAGAG cctgttgacaattaatcatcggca-3' and the reverse primer 5'-GAACTAATGACCCCGTAATTGATTACTATTAATAA CTAGTCTCATctcagcactgtcctgctcctt -3' introducing 45 nucleotides of flanking sequences. Subsequently, the GalK sequence was replaced with a dsDNA of the sequence actgcaaatttgatcaaacccagcttcagcttgcctgctccagagatgaga ctagttattaatagtaatcaattacggggtcattagttc resulting in deletion of ADP. To generate infectious virus, circular pKSB2-AdV-C2_dE3B_GFP_dADP was transfected into human embronic retinoblast 911 cells stably expressing I-SceI endonuclease (3), using the jetPEI transfection reagent (Polyplus transfection, Illkirch-Graffenstaden, France). Constitutive I-SceI expression in these cells was accomplished following transduction with MLV-ER-I-SceI-HA, which encodes a form of the endonuclease that can be translocated to the nucleus upon treatment with 4-OH-tamoxifen 3 h post transfection (4). I-SceI expression was confirmed by Western blotting of whole cell lysates using the α-HA antibody (HA.11 clone 16B12, Covance). Cells were selected in medium containing puromycin (1 μg / ml), and bulk cultures were expanded under selection conditions.

Supplementary References

1. **Georgi F, Kuttler F, Murer L, Andriasyan V, Witte R, Yakimovich A, Turcatti G, Greber UF.** 2020. High-content image-based drug screen identifies a clinical compound against cell transmission of adenovirus. BioRxiv. DOI: https://doi.org/10.1101/2020.05.15.098061
2. **Yakimovich A, Gumpert H, Burckhardt CJ, Lütschg VA, Jurgeit A, Sbalzarini IF, Greber UF.** 2012. Cell-free transmission of human adenovirus by passive mass transfer in cell culture simulated in a computer model. J. Virol. **86**:10123–10137.
3. **Ibanes S, Kremer EJ.** 2013. Canine adenovirus type 2 vector generation via I-Sce1-mediated intracellular genome release. PLoS One **8**:e71032.
4. **Courilleau C, Chailleux C, Jauneau A, Grimal F, Briois S, Boutet-Robinet E, Boudsocq F, Trouche D, Canitrot Y.** 2012. The chromatin remodeler p400 ATPase facilitates Rad51-mediated repair of DNA double-strand breaks. J. Cell Biol. **199**:1067–1081.
